# Emerging Approaches of Transcatheter Valve Repair/Insertion

**DOI:** 10.4061/2010/540749

**Published:** 2010-07-25

**Authors:** Maurizio Taramasso, Micaela Cioni, Andrea Giacomini, Iassen Michev, Cosmo Godino, Matteo Montorfano, Antonio Colombo, Ottavio Alfieri, Francesco Maisano

**Affiliations:** Cardiothoracic Department, San Raffaele Scientific Institute, via Olgettina 60, 20122 Milan, Italy

## Abstract

Aortic stenosis (AS) and mitral regurgitation (MR) account for the majority of valvular diseases and their prevalence is increasing according to increased life expectancy. Surgical treatment is the gold standard, although operative risk may be high in some patients due to comorbidities and age. A large part of the patients at high surgical risk who could beneficiate of treatment are not referred to surgery. Therefore, there is a need of alternative and less invasive procedures.

## 1. Introduction

Valvular heart disease is nowadays a relevant cause of morbidity and mortality. Aortic stenosis (AS) and mitral regurgitation (MR) account for the majority of valvular diseases and their prevalence is increasing according to phenomenon of population ageing [1, 2].

Surgery (aortic valve replacement for the AS and mitral valve repair or replacement for MR) is the gold standard treatment and warrants good and reproducible clinical and functional outcomes in most patients, although operative risk may be high in some patients due to comorbidities and age. Several registries revealed that up to 50% of the patients with severe valvular disease, even if a surgical indication existed, are not referred to surgery and the main reasons of this are a high operative risk, multiple comorbidities and advanced age [3, 4]. Thus, surgery is denied for a relevant number of patients who could beneficiate of it. Therefore, there is a need of alternative and less invasive procedures. The target of these novel minimal invasive techniques is to provide results similar to those of conventional surgery in terms of efficacy, safety, and durability. Over the past years several techniques for percutaneous repair and implantation have been developed. This paper provides an overview of these emerging approaches of transcatheter valve repair/implantation procedures.

## 2. Percutaneous Aortic Valve Therapy

Aortic stenosis is currently the most common valvular disease in the Western population, with a prevalence of 4.6% in adults ≥75 years [3, 5]. The need for aortic valve replacement (AVR) will continue to escalate according to the increase in life expectancy [6]. Surgical AVR has been performed since the early 1960s and is currently the standard treatment for patients with severe symptomatic AS, providing relief of symptoms and improving survival (survival has been shown to improve from 38% to 90% at 5 years following surgical AVR) and quality of life, even in the very elderly group [7–12]. Surgical AVR has an average operative mortality of 3% to 8%, with an important variability primarily due to patient characteristics, comorbidities, and reduced left ventricular function [7, 8, 13, 14]. Several scoring systems have been developed in order to help predict operative mortality, such as the logistic European System for Cardiac Operative Risk Evaluation (EuroSCORE) and the Society of Thoracic Surgeons (STS) score [15, 16].

Patients with high EuroSCORE or STS score are often denied surgery. The advent of transcatheter technologies may provide a desired alternative to medical management for these high-risk patients. 

Moreover, the feasibility of the implantation of a percutaneous aortic valve using the TAVI techniques (valve-in-valve concept) to treat patients with a degenerated bioprosthesis previously surgically implanted has been recently demonstrated [17, 18]. In the future, TAVI approach will likely become an option not just for elderly or high-risk patients, but also for younger patients with the knowledge that a valve-in-valve procedure can be performed if degeneration occurs. This could revolutionize the treatment of aortic valve disease. 

## 3. Current Techniques

First human implantation of a balloon-expandable percutaneous aortic valve (AV) was performed in 2002 by Cribier et al. to treat a severe calcified AS in a 57-year-old man with prohibitive surgical risk [19]. Since then, thousands of patients have undergone this procedure worldwide, and at this time, eight years after this initial experience, transcatheter aortic valve implantation (TAVI) is a primary therapeutic modality for high-risk or technically inoperable patients with critical AS.

In November 2007, a committee of experts including members of EACTS and ESC defined a consensus position statement for TAVI procedures [20].

Two different devices are under clinical investigation and commonly used for TAVI.

### 3.1. Edwards-Sapien Prosthesis

**Figure 1:**
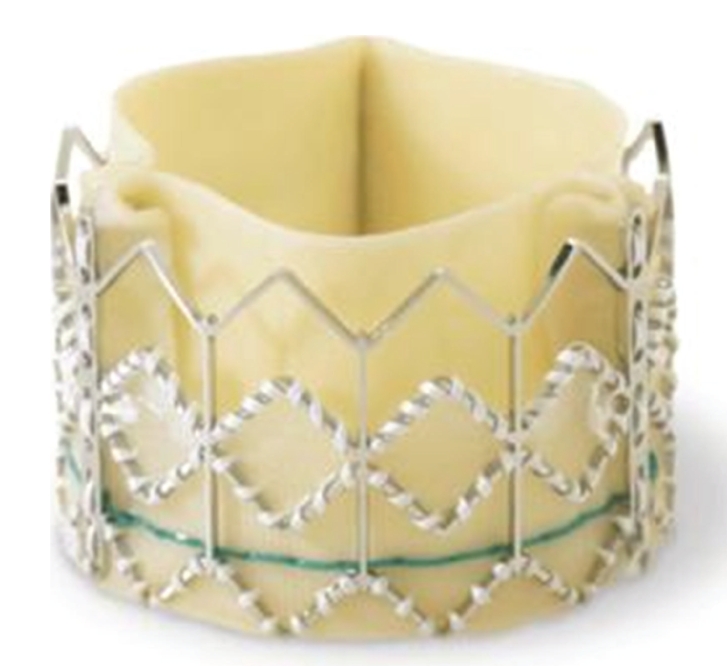
The Edwards-Sapien balloon-expandable prosthetic valve, constructed of a stainless-steel stent, bovine pericardial leaflets and a fabric sealing cuff.

The first device is the Edwards-Sapien valve (Edwards Lifescience, Inc., CA, USA—Figure 1). It consists of three bovine pericardial leaflets mounted within a balloon-expandable stainless-steel stent. Current prosthesis sizes include 23 and 26 mm. Current devices require either 22 F or 24 F (transfemoral) or 26 F (transapical) sheath for delivery [21]. 

The Edwards-Sapien valve was first implanted via the antegrade transseptal approach to the left atrium and passage through the mitral valve to reach the AV [19]. With this approach there is a high risk of anterior mitral valve leaflet injury, causing severe mitral regurgitation. Transfemoral retrograde approach has been shown to be safer and is now preferred [22–26]. Patients are usually placed under general anesthesia with endotracheal intubation, although sedation and analgesia may be sufficient. After crossing the AV, a balloon aortic valvuloplasty (BAV) is performed using standard techniques in order to predilate the stenotic valve. Simultaneous rapid right ventricular pacing using a temporary pacemaker (usually 180 beat/min), decreasing cardiac output, is used to stabilize the balloon during the inflation. The percutaneous valve is then placed within the aortic annulus, and when its position seems to be optimal, it is released. Also the deployment of the valve is achieved under rapid pacing. For the larger introducers (22 F, 24 F) used with the Sapien valves, two 10 Fr Prostar closure devices are usually placed within the vessel before any introducer sheath is sited. Because of the large delivery system, a surgical cutdown and repair of the vascular access site are still often required [27].

Because of the large profile of the device, many patients with small or diseased iliofemoral arteries are not eligible for the procedure. The minimal vessel diameter required for sheath insertion in the transfemoral approach is 7 and 8 mm for the 22- and 24-F catheters, respectively, for the Edwards-Sapien valve. 

An alternative transapical antegrade approach has been proposed, with good initial results [28–30]. Through a left anterolateral minithoracotomy, with the patient under general anesthesia, the pericardium is opened over the apex. Temporary pacing wires are placed on the left ventricle (LV), the LV apex is punctured, and 2 pledgeted sutures are placed. A stiff wire is passed to the descending aorta, and BAV is performed. The percutaneous valve is then deployed. For the transapical approach, femoral access and cardiopulmonary bypass should be on standby, to consent a rapid surgical conversion in case of complications.

### 3.2. CoreValve Revalving System

**Figure 2:**
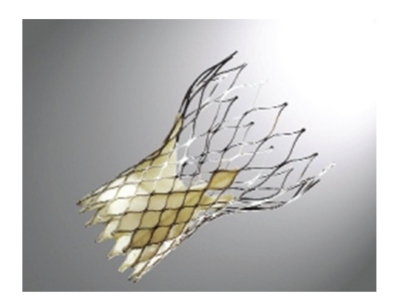
The CoreValve self-expandable prosthetic valve, constructed of a nitinol stent, pericardial leaflets, and sealing cuff.

The second device is the CoreValve Revalving System (Medtronic, Inc., MN, USA—Figure 2), which consists in three porcine pericardial leaflets mounted in a self-expanding nitinol frame. The CoreValve is available in 26 and 29 mm sizes, going through an 18 F introducer, allowing smaller arterial diameters (6 mm) in respect to the delivery system of the Edwards-Sapien valve. The total length of the valve is 50 mm. It has a specific design with a waist in the middle part. The lower part of the valve is designed to expand using high-radial forces. The middle part includes the leaflets and is constrained to avoid coronary occlusion, while the upper part enables fixation in the ascending aorta. The CoreValve is typically implanted retrograde from the femoral artery. 

A transaxillary approach has been proposed for the self-expandable valve and it has been used also with the balloon-expandable valve [31, 32]. 

Recently, for patients with “no-access options”, also a transaortic approach through a ministernotomy access has been described [33, 34]. The latest 3rd generation device enables performance of the procedure without rapid pacing [21, 24].

Whilst the Edwards system appears to have a greater requirement for native valve calcification in order to anchor the prosthesis, the CoreValve is self-expandable with a shape that seems less likely to embolize, even within a valve containing little calcium.

The positioning of the CoreValve is straightforward in theory; however, the self-expanding nature of the valve presents certain dynamism to the deployment process that remains, on occasion, challenging. An advantage of this device over the Edwards-Sapien valve, however, is that it is fully retrievable as long as the introducer catheter is not released from the valve. Once released, however, neither device is retrievable.

## 4. Patient Selection

### 4.1. Surgical Risk

TAVI is indicated in patients with pure or predominant severe AS, while the treatment of severe aortic regurgitation is still an off-label indication for TAVI. The most important issue in the selection of the candidates for TAVI is the analysis of the surgical risk and the evaluation of life expectancy. The decision-making process should involve a multidisciplinary team composed by cardiologists, cardiac surgeons, radiologists, and anesthesiologists. Physician's judgment in association with an assessment based on several surgical risks score (expected mortality > 20% with Logistic EuroSCORE and >10% with STS score) is the key element to establish patients at high surgical risk. This approach considers also factors that are not covered in the scores but that may make surgery prohibitive, such as porcelain aorta, previous CABG with patent graft, liver cirrhosis, or neurological dysfunction. Age alone or simply refusal of surgical intervention is not sufficient for indication to TAVI instead of surgery. TAVI should not be performed in patients presenting with a life expectancy <1 year because of extracardiac comorbidities [15, 16, 20, 35].

### 4.2. Anatomical Considerations

Once defined the surgical risk, feasibility of the TAVI procedure and exclusion of contraindications should be assess.

Coronary angiography or CT scan should be performed to evaluate the anatomy of the coronary arteries. However, many of these patients have calcified coronary disease, limiting the value of CT scan. In case of need of revascularization, the chronology and the modality of interventions should be individualized based on the clinical status of the single patients.

The correct sizing of the AV is the critical step to establish the feasibility of the TAVI and to minimize the risk of paravalvular leak or prosthesis migration after the deployment. A gold standard method of measurement has not been yet established. Echocardiography and CT scan are both used [36–40]. 

The evaluation of the state of the peripheral access (size, tortuosity, and calcification) is obtained by angiography or CT scan [20, 41].

The most important technical contraindications for TAVI are the inadequacy of the aortic annulus (<18 mm or >25 mm for balloon-expandable prosthesis and <20 mm or >27 mm for self-expandable), presence of asymmetric valvular calcification (because of the high risk of compression of the coronary arteries), an ascending aortic dimension >45 mm at sinotubular junction for the self-expandable valve and the presence of LV thrombosis [20, 23].

The choice of the correct approach (transfemoral or transapical) should be discussed according to the patient condition and local expertise. Specific contraindications for transfemoral approach include inadequacy of femoro-iliac arteries (severe calcification, tortuosity, or small diameter, according to the device used or previous aorto-femoral bypass), transverse ascending aorta for the balloon-expandable device, and adverse aortic condition (severe angulation, atheroma of the arch, and aneurysm of abdominal aorta with protruding mural thrombus). Contraindications for transapical approach are previous LV surgery (such as Dor procedure), severe respiratory insufficiency, not reachable LV apex through thoracotomy, and calcified pericardium [20].

In patients presenting with an estimated extreme clinical risk or because of anatomical inadequacy for TAVI, a BAV procedure may be useful as a bridge to subsequent percutaneous implantation or as palliation. No survival benefit has been showed after BAV [1–3, 42].

## 5. Results

Procedural success exceeds 90% in experienced centers. Mortality at 30-days ranges from 5% to 18% [22–26, 43–45]. The 30 day mortality reported in the SOURCE Registry, which was designed to assess the initial clinical results of the Edwards-Sapien valve in consecutive high-risk patients in Europe, was6.3%in TF patients and 10.3% in TA patients [46, 47].The recently published Canadian experience with the Sapien valve reported an overall 30-day mortality of 10.4% (9.5% for TF approach and 11.3% for TA approach) and a mortality rate at a mean followup of 8 months of 22.1% [44].

Incidence of stroke is 1.7%–2.5% and it seems to be similar for both procedural approaches [44, 46].

The periprocedural death observed with the CoreValve Revalving System is similar [43].

Vascular complications remain a significant cause of mortality and morbidity (incidence 10%–15%) [23, 44–46], also if in the SOURCE Registry this type of complication was proved to be no longer a predictor of <30-day mortality in the transfemoral approach [46]. Acute myocardial infarction occurs in 2%–5% [46]. Coronary obstruction and prosthesis embolization are rare (<1%) [20, 24, 26, 48]. Mild-to-moderate AR, without hemodynamic impairment, is observed in about 50% of the patients, mostly para-valvular. Significant AR occurs in 2%–5% [24, 26, 43]. Finally, AV block occurs in 4%–10%, necessitating pacemaker implantation in up to 24% with self-expandable device [49–53]. Left bundle-branch block with left-axis deviation, interventricular septal dimension >17 mm, and noncoronary cusp thickness >8 mm were identified as predictors of AV block and need of permanent pacemaker following placement of self-expandable prosthesis [54].

## 6. Our 2-Year Experience with TAVI in High-Risk Surgical Candidates

From November 2007, 137 patients with severe aortic stenosis underwent TAVI at our Institution, 64 females with mean age 79 ± 7 years. Peak and mean aortic gradients were 88.2 ± 25.4 mmHg and 53.9 ± 15.7 mmHg, respectively. Mean Logistic EuroSCORE and STS-PROM score were 26.3 ± 16 and 7 ± 4.9, respectively. Patients had multiple comorbidities (Charlson score 6 ± 1.7). Baseline characteristics of the patients are shown in Table 1. 

An Edwards-Sapien valve was implanted in 79 patients (61 transfemoral, 15 transapical; and 3 transaxillary); a CoreValve was implanted in 58 patients (46 transfemoral and 12 transaxillary). Procedural success rate was 99.2% (1 acute aortic dissection requiring emergent surgery). Hospital mortality (30 days) was 3%. Postoperative complications included vascular lesions (62 patients), renal failure (10 patients), need for PM (38 patients), moderate to-severe-aortic regurgitation (14 patients), and cerebrovascular event (6 patients). Mean length of stay was 9 ± 9.8 days (range 2-to-68 days) (see Table 2). Followup was 100% complete (mean 6 ± 4 months). Actuarial 6-month survival was 89% ± 5% for the combined experience and it was 73% ± 16%, 100%, and 91% ± 4% for the transapical, transaxillary and transfemoral approaches, respectively (Tables 2 and 3). 

## 7. Percutaneous Mitral Procedures for the Treatment of Mitral Regurgitation

The development and evaluation of mitral valve (MV) repair technologies represents an emerging challenge. Currently, several percutaneous approaches for the treatment of MR are in early clinical use or undergoing preclinical investigation. Percutaneous repair of the MV has many differences compared to TAVI procedures, in consideration of the different mechanisms that can lead to MR (variable annular dilatation, abnormal leaflet coaptation, abnormal chordal structure, among others). One or more of the elements of the MV apparatus may be involved according to the etiology of the MR and the patient population may be very heterogeneous for age and comorbidities [55]. 

**Figure 3:**
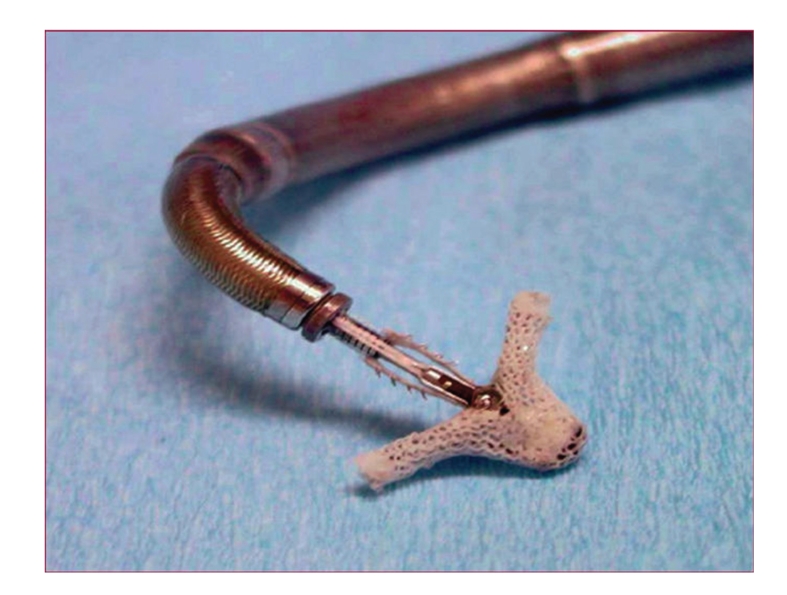
The Mitraclip device is a two-armed, polyester-covered, fixation device. Each arm has an opposing gripper that aids the leaflets in the clip by means of multipronged friction element.

The most advanced percutaneous MV repair procedure is the Alfieri edge-to-edge repair using the Evalve Percutaneous Mitral Repair System, also known as MitraClip device (Evalve, Inc., CA, USA—Figure 3). The Alfieri repair consists in suturing the free edge of the anterior mitral leaflet to the free edge of the posterior leaflet at the site of the regurgitation. The result is a double-orifice valve with improved leaflet coaptation. This surgical procedure has proven early efficacy and durability in various anatomic and functional lesions [56–58]. The Mitraclip system reproduces the surgical procedure using a clip to join the free edges of the opposing leaflets [59]. This procedure involves transseptal cannulation of the left atrium and positioning the delivery system perpendicular to the MV. Under TEE guidance, the clip is placed to appose the two mitral leaflets, creating a double-orifice valve. The reduction of the MR severity is real-time assessed and, if necessary, it may be repositioned to reduce MR further. The operator should be familiar with echo imaging and a close collaboration between the operator and the echocardiographist is mandatory to run the procedure safely and efficiently. Live 3D echocardiography is very helpful particularly for clip orientation and alignment on the coaptation line. Final echo assessment should be done under vasoconstrictors. When the result is satisfactory, the clip is deployed and the delivery system is removed. In case of persistent significant MR (>2+/4+), a second clip may be placed. 

Recently, the feasibility of the procedure using conscious sedation has been reported in [60].

## 8. Patient Selection

**Figure 4:**
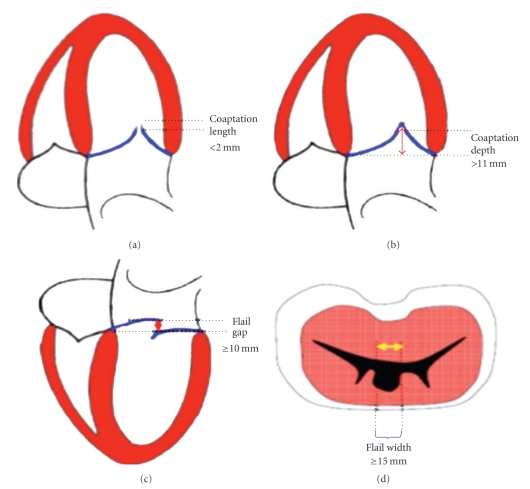
Anatomical exclusion criteria for the percutaneous edge-to-edge repair.

Patient selection is fundamental for the efficacy of the procedure. Indications for the Mitraclip are still preliminary and will continue to evolve as the techniques and technologies will prove efficacy and safety. The best indication for the endovascular edge-to-edge is given in those patients with severe degree of mitral regurgitation and with symptoms that are at high risk for surgery. From a pure technical standpoint, the procedure is feasible only in a subgroup of patients with specific anatomical characteristics and this makes the eligibility limited (see Figure 4).

The first step in the screening process to select patients who could be eligible for the procedure is transthoracic echocardiography, especially with regard to the parasternal short-axis view of the mitral valve and origin of MR jet(s). The main eligibility criterion is jet location.

Definitive patient selection is done by transesophageal echocardiography. Mitral regurgitation should originate from the middle of the valve (from A2–P2 segments). The mechanism of regurgitation can be either a prolapse or a restricted motion not related to rheumatic disease. The discontinuation between the two leaflets at the site of regurgitation should be minimal and the annular dilatation and/or calcification should be absent or non relevant. In case of prolapse, the jet width should be less than 15 mm, and the flail gap less than 10 mm. In case of functional MR, the jet width should be again less than 15 mm, and the coaptation depth ideally should be less than 10 mm, with the leaflets having a minimal residual coaptation [61–63].

## 9. Results

The results phase I of the multicenter prospective trial EVEREST (endovascular valve edge-to-edge repair study), including 107 patients with central MR (21% with pure functional MR) who were eligible for surgical MV repair showed a periprocedural incidence of major events of 9%, including 1 nonprocedural death. Freedom from clip embolization was 100%. Procedural success was achieved in 74% of the patients, and 64% presented MR of≤1+at discharge. Followup data showed that surgical option after percutaneous repair was preserved (32 patients had mitral valve surgery during the 3.2 years after clip procedures; in case of planned mitral repair, successful repair was obtained in 84%) [64]. 

Among the successfully treated patients, freedom from death, mitral valve surgery, and MR >2+ at 12 months were 66%. Overall survival was 90.1%, at 3 years. No differences were observed in terms of acute results and durability between patients with degenerative and functional MR [64].

The results of phase II of the study (EVEREST II), which compared standard surgical repair to percutaneous edge-to-edge mitral repair (randomization 2 : 1 MitraClip to surgery), showed an incidence of major adverse events at 30 days (death, stroke, reoperation of MV, urgent/emergent cardiovascular surgery, myocardial infarction, renal failure, deep-wound infection, ventilation >48 hours, new onset permanent atrial fibrillation, septicemia, gastrointestinal complication requiring surgery, and transfusions ≥2 units) of 9.6% for the clip and of 57% for surgical MV repair. Clinical success rate (freedom from death, MV surgery or re-operation for MV dysfunction, and MR >2+) at 12 months was 72% for the clip and 88% for surgical MV repair [65]. These results confirmed that the MitraClip procedure is a safe and effective therapeutic option for selected patients with significant MR.

## 10. Our Initial Experience in High-Risk Patients

In our Institution, 28 patients with severe MR have been treated using the Mitraclip. 22 patients had functional MR (FMR) and 6 patients had degenerative MR. Procedures were performed under general anesthesia, using live 3D transesophageal echocardiography. Mean age was 67.9 ± 14.4 years and mean EF was 35% ± 20% (25% ± 7% in FMR patients). All patients had severe central MR, dilated ventricles (mean DTD 67 ± 6 mm), and pulmonary hypertension (PAPs was 45 ± 21 mmHg). Mean logistic EuroSCORE was 23.9 ± 15.5. In 17 patients there was the need of a second clip. Median stay in the ICU and in the general ward was 1 and 5 days, respectively. Postprocedural course was complicated by low cardiac output requiring inotropes in 6 patients (21.4%), acute renal insufficiency in 3 patients (10.7%), hemopericardium in 1 patient (3.5%), need of IABP in 2 patients (7.1%), and need of hemotransfusions in 3 patients (10.7%). At discharge, 2 patients (7.1%) had no MR (0+/4+), 21 patients (75%) had mild (1+/4+) MR, and 5 patients (17.8%) had moderate (2+/4+) MR.

## 11. Other Percutaneous MV Repair Procedures

Several alternative percutaneous procedures for the treatment of the MV are being evaluated in preclinical studies. 

The coronary sinus annuloplasty is based on the close anatomical relation of the coronary sinus with the posterior mitral annulus, and several devices exist for this approach: the aim is to place devices in the coronary sinus to push against the posterior portion of mitral annulus, in order to improve the coaptation of the leaflets. Although success in animals has been observed, human trials have presented more difficulties, likely because often the coronary sinus, which is an atrial structure, is not in the same plane as the mitral annulus. Moreover, the circumflex coronary artery or its branches may lie between the mitral annulus and the coronary sinus, and the distance between the coronary sinus and the posterior mitral annulus increases with chronic ischemic MR because of the ongoing remodeling process [66–68].

The most promising coronary sinus annuloplasty device is the CARILLON Mitral Contour System (Cardiac Dimensions, Inc., Kikland, Wash), which uses 2 self-expanding nitinol anchors connected by a wire: the distal coronary sinus anchor is deployed, then manual tension is applied to the connecting wire and the proximal anchor is deployed, obtaining a shortening of the mitral annular dimension. The tension on the device can be adjusted before the final release. The multicenter AMADEUS trial suggested an acute reduction in MR by a mean of 1 grade with the modified CARILLON XE device [69, 70].

Other techniques are based on the concept of moving the ventricle, rather than the annulus, to increase leaflet coaptation and eliminate functional MR. The Coapsys device (Myocor, Inc., MN, USA) employs a transventricular splint with pads on the outer surface of the left ventricle. In an open chest, this can be placed without cardiopulmonary bypass under direct echocardiographic guidance. Pads attached to each end of the splint are tightened to pull the ventricle into the region of the papillary muscles and also to move the posterior leaflet to better coapt with the anterior leaflet. Initial studies with the open-chest Coapsys system showed encouraging results [71–73].

The i-Coapsys device performs the same role but can be delivered in a minimally invasive procedure with fluoroscopic guidance [74].

Another field of application of percutaneous procedures for MR is the correction of a paravalvular leak following surgical MV replacement. When the periprosthetic leak is haemodynamically relevant, a percutaneous treatment may offer an alternative to the redo surgery in high-risk patients. Several different types of devices that have not been specifically designed for this purpose may be used (usually coils for very small defects, patent ductus devices for medium defects and atrial septal occluders for large defects). Results of transcatheter mitral paravalvular leak have achieved variable success rates, with a reported initial success rate of 60% to 90% and a need for repeat intervention of about 40% [21, 75–77]. 

The use of advanced image guidance with 3D echocardiography could enhance the technical success rate. The most important limitation remains the use of preexisting non specific devices.

## 12. Limitations and Potential of Percutaneous Treatment of Heart Valve Disease

With regard to TAVI, the pending questions concern mainly safety and long-term durability. Using it in patients who are good surgical candidates seems to be still premature. Thus, TAVI should currently be restricted to patients at high surgical risk or with contraindications for conventional surgery. The emerging concept of valve-in-valve may provide a feasible treatment option if and when currently implanted percutaneous aortic valves deteriorate. Moreover, with the advent of TAVI procedures, a degenerated bioprosthesis can now be replaced less invasively and perhaps with less risk using the transcatheter valve implantation techniques. Therefore, current guidelines for choosing the type of valve prosthesis may indeed require a revision.

With regard to MV repair using the Mitraclip, the surgical experience with Alfieri edge-to-edge technique indicates that significant MR recurs if the procedure is not associated to an annuloplasty (need for reoperation 30% at 5 years), especially in presence of a dilated mitral annulus [78–80], so the long-term results of this technique have still to be evaluated. 

Percutaneous MV repair is attractive and should be considered in all patients with functional MR (ischemic or dilatative) at high surgical risk due to advanced age, concomitant comorbidities, or advanced heart failure, especially in presence of depressed EF, right ventricular dysfunction, or severe pulmonary hypertension. The main obstacles to the extension of this technique in high-risk patients are the limited anatomical eligibility and the long learning curve for the operators.

Another emerging field of application of percutaneous MV repair is the correction of simple degenerative MR in young patients. The goal of modern MV valve surgery, in fact, is to obtain excellent long-term results using an approach as little as possible invasive, such as miniinvasive or robotic surgery. The main purpose is the attempt to totally neutralize the disease, obtaining a survival and a quality of life comparable to an age-matched population. If the operation is performed by conventional surgery, for example, in a young female patient, the disease cannot be considered neutralized, because of the persistence of the scar: neutralizing the disease concerns also this aspect. This consideration may justify the use of percutaneous MV repair in young patients with simple MV prolapse, providing that percutaneous edge-to-edge does not preclude surgical mitral repair in case of failure of the percutaneous repair [81, 82]. As long as the percutaneous procedure is safe, even if success rate is lower than that of surgery, patients with failed percutaneous repairs are still candidates for surgical repair. Therefore, transcatheter therapy may temporarily delay or avoid the need for surgical intervention.

## 13. Conclusions

Transcatheter aortic and mitral valve procedures are evolving very rapidly and are currently a therapeutic modality for the patients with severe valvular disease who are unsuitable for surgery because of technical/anatomical issues or too high-estimated surgical risk. Several clinical trials, including randomized studies between surgical and percutaneous treatment, are currently ongoing and in the near future the indications for these procedures likely will be extended to patients who are good candidates for surgery.

## Figures and Tables

**Table 1:** Clinical characteristics of patients presenting for transcatheter aortic valve implantation.

	Transfemoral	Transapical	Transaxillary	*P* value
Patients	107	15	15	
Edwards-Sapien, *n* (%)	61 (57)	15 (100)	3 (20)	<.0001
CoreValve, *n* (%)	46 (43)		12 (80)	<.0001
Age (years)	79.7 ± 7	78.8 ± 6.5	78.7 ± 5	.794
Male, *n* (%)	56 (52)	5 (33)	12 (80)	.034
Diabetes, *n* (%)	31 (29)	4 (26.7)	5 (33)	.917
Chronic kidney disease, *n* (%)	39 (36.4)	5 (33)	7 (46.7)	.706
Porcelain aorta, *n* (%)	17 (15.9)	14 (93.3)	7 (46.7)	<.0001
COPD, *n* (%)	46 (43)	6 (40)	11 (73)	.077
Previous myocardial infarction, *n* (%)	29 (27)	5 (33)	6 (40)	.549
Cerebrovascular disease, *n* (%)	20 (18.7)	8 (53)	3 (20)	.011
Peripheral vascular disease, *n* (%)	26 (24.3)	10 (67.7)	12 (80)	<.0001
NYHA functional class III-IV, *n* (%)	75 (70)	11 (73.3)	9 (60)	.685
Logistic EuroSCORE, mean ± SD	26.6 ± 16	32.2 ± 23	28.6 ± 14	.477
STS-PROM score, mean ± SD	7 ± 4.9	8.3 ± 4.2	6.9 ± 2.8	.602
Mean aortic gradient (mmHg), mean ± SD	54 ± 17.2	44.7 ± 18	47.7 ± 14.9	.074
LVEF (%), mean ± SD	50.8 ± 12.9	50 ± 12.5	52.8 ± 11	.814

COPD: chronic obstructive pulmonary disease; NYHA: New York Heart Association; LVEF: left ventricle ejection fraction.

**Table 2:** Procedural and in-hospital results of patients submitted to transfemoral TAVI approach in our Institution.

Patients (*n*)	**107**
Hospital stay, days (mean ± SD)	9.5 ± 1.4
Procedural success, *n* (%)	100 (93.5)
Vascular complications, *n* (%)	31 (29)
Need for permanent pacemaker, *n* (%)	19 (17.8)
Neurological event, *n* (%)	3 (2.8)
Acute renal failure requiring CVVH, *n* (%)	5 (4.7)

*30-day clinical outcomes*	
Death, *n* (%)	1 (0.9)
Cardiac death, *n* (%)	0
LVEF, *n* (%)	52.6 ± 11.5

*Six months cumulative clinical outcomes*	
Death, *n* (%)	12 (12.2)
Cardiac death, *n* (%)	2 (2.0)
LVEF (%), mean ± SD	53.22 ± 9.1

SD: standard deviation; CVVH: continuous veno-venous hemofiltration; LVEF: left ventricle ejection fraction.

**Table 3:** Procedural and in-hospital results of patients submitted to trans-apical and trans-axillary TAVI Approach in our Institution.

	Trans-apical	Trans-axillary
Patients (*n*)	15	15
Hospital stay (days), mean ± SD	15.8 ± 4.8	8.7 ± 1
Procedural success, *n* (%)	13 (86.6)	14 (93.3)
Need for permanent pacemaker, *n* (%)	3 (20)	1 (6.7)
Neurological events, *n* (%)	1 (6.7)	1 (6.7)
Acute renal failure requiring CVVH, *n* (%)	4 (26.6)	2 (13.3)

*30-day clinical outcome*		
Death, *n* (%)	2 (13.3)	0
Cardiac death, *n* (%)	2 (13.3)	0
LVEF (%), mean ± SD	49.67 ± 10.4	52.73 ± 10.1

*Six-month cumulative clinical outcome*		
Death, *n* (%)	4 (26.6)	2 (18.2)
Cardiac death, *n* (%)	4 (26.6)	1 (9.1)
LVEF (%), mean ± SD	56.22 ± 4.12	60.23 ± 3.45

SD: standard deviation; CVVH: continuous venovenous hemofiltration; LVEF: left ventricle ejection fraction.
